# Cd248a and Cd248b in zebrafish participate in innate immune responses

**DOI:** 10.3389/fimmu.2022.970626

**Published:** 2022-08-31

**Authors:** Xianpeng Li, Ruitong Guo, Shuaiqi Yang, Xiangmin Zhang, Xiu Yin, Lei Teng, Shicui Zhang, Guangdong Ji, Hongyan Li

**Affiliations:** ^1^ College of Marine Life Sciences, and Institute of Evolution & Marine Biodiversity, Ocean University of China, Qingdao, China; ^2^ Department of Genetics and Cell Biology, Basic Medical College, Qingdao University, Qingdao, China; ^3^ Laboratory for Marine Biology and Biotechnology, Pilot National Laboratory for Marine Science and Technology (Qingdao), Qingdao, China

**Keywords:** zebrafish, cd248a, cd248b, immune, LPS, inflammatory cytokines

## Abstract

CD248, also known as endosialin or tumor endothelial marker 1, is a type I single transmembrane glycoprotein. CD248 has been demonstrated to be upregulated in cancers, tumors and many fibrotic diseases in human and mice, such as liver damage, pulmonary fibrosis, renal fibrosis, arthritis and tumor neovascularization. However, no definite CD248 orthologs in fish have been documented so far. In this study, we report the identification of *cd248a* and *cd248b* in the zebrafish. Both the phylogenetic analysis and the conserved synteny strongly suggested that zebrafish *cd248a* and *cd248b* are orthologs of the human *CD248*. Both *cd248a* and *cd248b* exhibited similar and dynamic expression pattern in early development, both genes had weak maternal expression, the zygotic transcripts were first seen in anterior somites and head mesenchyme, then shifted to eyes and head mesenchyme, later expanded to branchial arches, and gradually declined with development. The expression profiles of *cd248a* and *cd248b* were upregulated upon LPS (Lipopolysaccharide) challenge. Both Cd248a protein and Cd248b protein were localized on the cell membrane and cytoplasm, and overexpression of *cd248a* and *cd248b* induced the expression of pro-inflammatory cytokines, *in vitro* and *in vivo.* Moreover, deficiency of *cd248a* or *cd248b* both downregulated the expression of pro-inflammatory cytokines and upregulated anti-inflammatory cytokine. Additionally, loss of *cd248a* or *cd248b* both downregulated the expression of pro-inflammatory cytokines after LPS treatment. Taken together, these results indicated that *cd248a* and *cd248b* in zebrafish were involved in immune response and would provide further information to understand functions of Cd248 protein in innate immunity of fish.

## Highlights

1. Zebrafish *cd248a* and *cd248b* are orthologs of human *CD248.*
2. Zebrafish *cd248a* and *cd248b* exhibited similar expression pattern, predominantly on the anterior somites, head mesenchyme, eyes and branchial arches.
3. Zebrafish Cd248 proteins participate in immune response.

## Introduction

CD248, also known as endosialin or tumor endothelial marker 1, is a type I single transmembrane glycoprotein (1–3). CD248 was initially identified as an additional tumor stromal antigen (FB5) in human (4). CD248 is a member of C-type lectin domain group 14 family that includes CLEC14A, CD93 and thrombomodulin (also known as TM or THBD), which contain C-type lectin domain (5, 6).

Contrary to the early reports, CD248 is not expressed by endothelial cells, but mainly expressed in stromal fibroblasts, myofibroblasts and a subset of pericytes (1–3). CD248 is expressed in early embryos and is almost undetectable in normal adult mouse tissues except low level in fibroblasts and pericytes (3). CD248 has been shown to be upregulated in cancers, tumours, and many fibrotic diseases, and is considered as a therapeutic target and biomarker (7, 8). It has been shown CD248 participates in angiogenesis, hypoxic regulation, inflammation and reorganization of the extracellular matrix. Vascular smooth muscle cells played a key role in the pathogenesis of atherosclerosis, while CD248 promoted atherosclerosis by remodeling the phenotype of vascular smooth muscle cells (9). Moreover, CD248 expressed in hepatic stellate cells played a key role in regulating the balance between liver fibrosis and hepatocyte proliferation during liver injury, thus endosialin was identified as a therapeutic target in non-neoplastic settings (10). Interestingly, hypoxia, inflammation, and fibrosis in obesity could be eliminated by adipocyte-specific CD248 knockdown, which makes CD248 a potential target for improving metabolic health (11). In addition, CD248 is also involved in renal fibrosis (12), pulmonary fibrosis (13), arthritis (14, 15), and tumor neovascularization (16, 17), and has become a potential target for the treatment of these diseases. Furthermore, CD248 is involved in the immune response. CD248 is expressed on the mesenchymal stromal cells in thymus plays an important role in thymus development and thymus regeneration after infection, further participates in peripheral immunity (18). CD248 also plays an important role in the remodeling of secondary lymphoid organs during adaptive immunity (19). In addition, CD248 expressed by naive human CD8+ T cells inhibits the proliferation of T-cells (20).

CD248 has been extensively studied in humans and mice, but the information regarding *cd248* in lower vertebrate is lacking and the role of Cd248 in lower vertebrate is still undefined. In the present study, we identified the orthologs of *cd248* in zebrafish, referred as *cd248a* and *cd248b*. In brief, we analyzed the expression pattern in early embryos and upon LPS challenge, we also investigated the effect of *cd248* on the expression of pro-inflammatory cytokines. Deficiency of *cd248a* or *cd248b* downregulated the pro-inflammatory cytokines. In addition, loss of *cd248a* or *cd248b* both downregulated the expression of pro-inflammatory cytokines after LPS treatment. Collectively, our data suggested that zebrafish Cd248 proteins were involved in immune response.

## Materials and methods

### Zebrafish maintenance and cell culture

The AB strain zebrafish (*Danio rerio*) were maintained at 28 ± 1°C and fed twice daily under 14 hours for light and 10 hours for dark cycle. The embryos were produced by natural mating. The experiments were performed according to the regulations and guidelines issued by Animal Committee of Ocean University of China.

HEK293T cells and RAW264.7 (leukemia cells in mouse macrophage) cells were cultured in Dulbecco’s modified Eagle’s medium (DMEM) containing 10% fetal bovine serum (FBS) in a 37˚C humidified incubator with 5% CO_2_ environment. Cell transfections were carried out using Lipofectamine 2000 following the manufacturer’s instructions.

### Bioinformatics analysis

The orientation and location of all genes used for gene synteny were referenced on the NCBI website. The protein domains were predicted using SMART website (http://smart.embl.de/).

Phylogenetic tree was constructed by neighbor-joining method and maximum-likelihood method with 1000 bootstrap replicates using MEGA 7.0 (21). All of the amino acid sequences used in bioinformatics analysis were downloaded from NCBI website (https://www.ncbi.nlm.nih.gov/).

### Whole-mount in suit hybridization

The fragments of *cd248a* and *cd248b* were amplified by PCR using the specific primers P5、P6 and P7、P8, respectively. Both fragments were digested by *Nco I* (Promega, USA) and then Digoxigenin (DIG)-labeled antisense probe were synthesized *in vitro* by Sp6 RNA polymerase. The procedures of WISH were following the protocol (22). The staining embryos were observed and photographed under the stereomicroscope (Nikon, Japan).

### Microinjection of LPS and quantitative real-time PCR

An aliquot of 6 nL PBS containing 6×10^-3^ ng LPS (treatment group) or PBS alone (control group) was injected individually into each yolk sac of zebrafish 1-cell stage embryos (23), and two groups were cultured separately in thermostat incubator. At 0 (not injection), 2, 4, 6, 10, 12, 14, 24, 48, 72, 96 and 120 hours post-injection, 40 embryos/larvae were sampled at each stage in each group, and homogenized in Trizol Reagent. Total RNAs extraction and purification using Total RNA Kit I (Omega) according to the manufacturer’s instructions, then were synthesized to cDNA by reverse transcription system (Takara). The expression of *cd248a* and *cd248b* mRNA after LPS injection was analyzed by qRT-PCR using ABI 7500 real-time PCR system (Applied Biosystems). The specific primers of *cd248a* (P9、P10) and *cd248b* (P11、P12) for qRT-PCR to detect the expression level of the relative mRNA, zebrafish *β-actin* (P13、P14) gene was chosen as the reference for internal standardization. qRT-PCR reaction conditions were as follows: 95°C for 15s, followed by 40 cycles of 95°C for 5s, 60°C for 15s, and 72°C for 35s. Each experiment was repeated three times. Data were quantified with the comparative Ct method (2^-(△△ct)^) in order to calculate the relative mRNA expression level (23). The data obtained from qRT-PCR analysis were subjected to two-way analysis of variance (ANOVA) to determine differences in the mean values. Data were shown as mean ± SD. *P < 0.05; **P < 0.01; ***P < 0.001; ****P < 0.0001.

### Subcellular localization

The *pcDNA3.1/V5/eGFP* vector was formed by cloning *eGFP* gene into eukaryotic expression vector *pcDNA3.1/V5-His A*. Subsequently, the complete coding regions of *cd248a*, *cd248b*, *cd248b_ΔN_* (lack of N-terminal transmembrane region), *cd248b_ΔC_* (lack of C-terminal transmembrane region) and *cd248b_ΔNΔC_* (lack of N- and C-terminal transmembrane regions) were amplified by PCR using the specific primers (P1、P2, P3、P4, P5、P4, P3、P6 and P5、P6 respectively) containing restriction enzyme cutting sites *EcoR I* and *Xho I*, and then the fragments were inserted into the upstream of *pcDNA3.1/V5/eGFP* to construct the recombinant plasmids. To examine subcellular localization of *cd248a* and *cd248b* and its variants in HEK293T, the *pcDNA3.1/V5/cd248a/eGFP*, *pcDNA3.1/V5/cd248b/eGF*, *pcDNA3.1/V5/cd248b_ΔN_/eGFP*, *pcDNA3.1/V5/cd248b_ΔC_/eGFP*, *pcDNA3.1/V5/cd248b_ΔNΔC_/eGFP* and *pcDNA3.1/V5/eGFP* recombinant plasmids were transfected individually into HEK293T cells as described above. At 24h post-transfected, the cells were stained with DAPI. After washing with PBS, the samples were observed from confocal microscope.

### Effect of overexpression of zebrafish cd248a and cd248b on pro-inflammatory cytokines expression in mouse macrophages

The open reading frames of *cd248a* and *cd248b* were inserted separately into the eukaryotic expression vector *pcDNA3.1/V5-His A* by *EcoR I* and *Xho I* restriction enzyme, and the specific primers used to clone the fragments of *cd248a* and *cd248b* were P15、P16 and P17、P18, respectively. Then *pcDNA3.1/V5/cd248a*, *pcDNA3.1/V5/cd248b* and *pcDNA3.1/V5-His A* plasmids were transfected individually into RAW 264.7 cells. At 24h after transfection, the total RNA of the cells was extracted and cDNA was synthesized. Finally, we examined the expression levels of mouse *Tnfα* (P19 and P20), *Il1β* (P21 and P22) and *Il6* (P23 and P24) by qRT-PCR. Mouse *β-actin* (P25、P26) gene was chosen as the reference for internal standardization (24). Each experiment was repeated three times.

### Effect of overexpression of zebrafish cd248a and cd248b mRNA on pro-inflammatory cytokines expression *in vivo*

To further verify the effect of overexpression of zebrafish *cd248* on pro-inflammatory cytokines *in vivo*, we synthesized *cd248a* and *cd248b* mRNA by SP6 mMESSAGE mMACHINE Kit (ThermoFisher). Diluted mRNAs were injected into 1-cell stage embryos of zebrafish. At 48h after injection, the total RNA of the embryos was extracted and cDNA was synthesized. Finally, we examined the expression levels of *tnfα* (P27 and P28), *il1β* (P29 and P30), *il6* (P31 and P32), *hif1a* (P33 and P34), *pigf* (P35 and P36), *vegfr1* (P37 and P38), *notch3* (P39 and P40) and *mmp9* (P41 and P42) by qRT-PCR. Zebrafish *β-actin* gene was chosen as the reference for internal standardization (24). Each experiment was repeated three times.

### Effect of cd248 deletion on pro-inflammatory cytokines and anti-inflammatory cytokine

The mutants of *cd248a* and *cd248b* were carried out by CRISPR/Cas9 system (25). The sequences for gRNAs were shown in **Figures 8A–F**. At 48h after fertilization, we extracted the total RNA of WT and mutant embryos and synthesized cDNA. Finally, we examined the expression level of *cd248a*, *cd248b*, *tnfα*, *il1β*, *il6* and *il4* (P43 and P44) by qRT-PCR as previously described. These pro-inflammatory cytokines were also examined after LPS treatment. Each experiment was repeated three times.

### Supplementary data

All primers used in this paper were shown in **Supplemental Table 1**. All protein sequences used to construct phylogenetic trees were shown in **Supplemental Table 2**.

## Results

### Two orthologs of the human CD248 were present in the zebrafish genome

**Figure 1:**
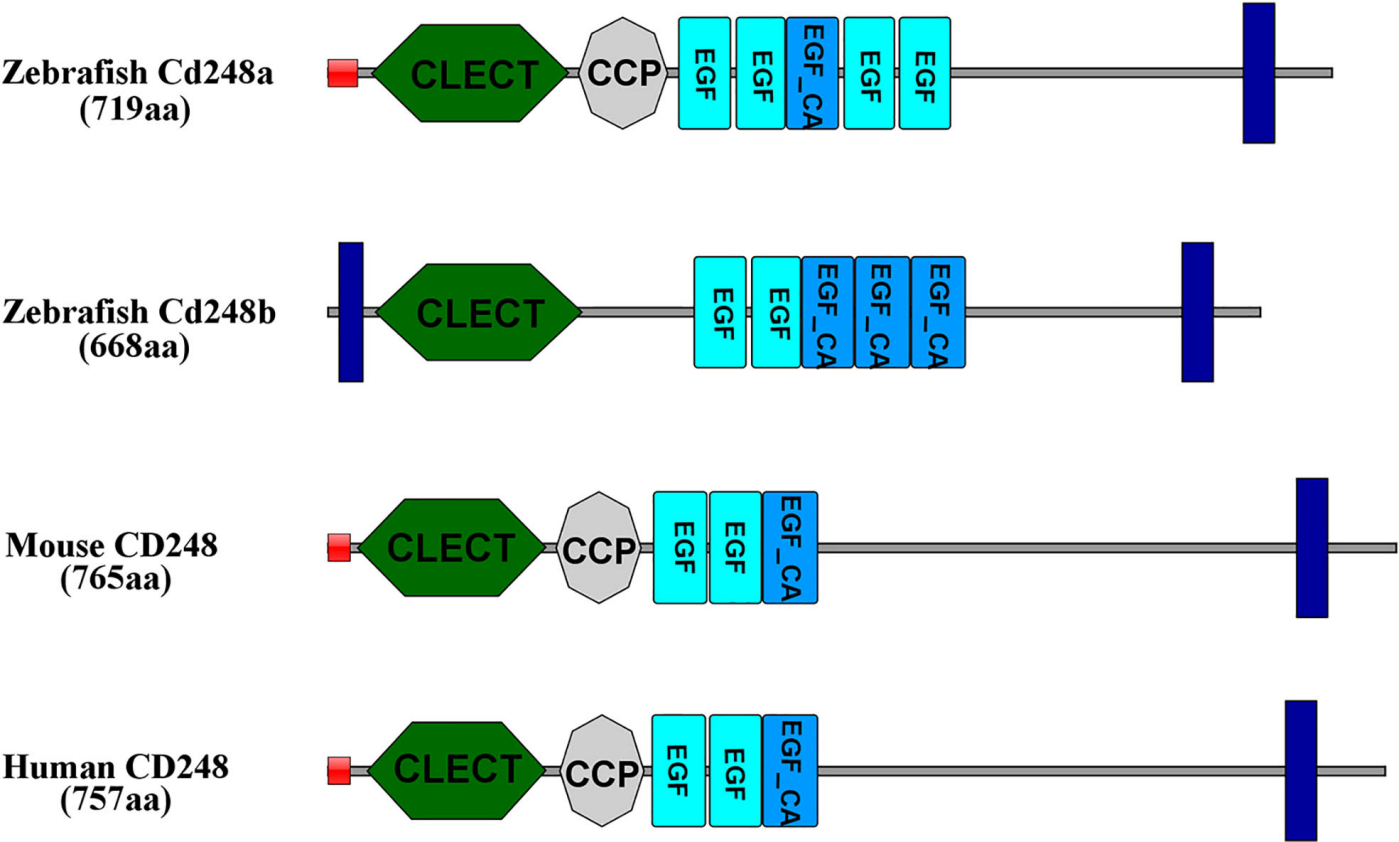
Amino acid functional domains of CD248 protein in human, mouse and zebrafish. Red squares, signal peptide; Blue rectangles, transmembrane region; CLECT, C-type lectin domain; EGF, epidermal growth factor-like domain; EGF_CA, Calcium-binding EGF-like domain; CCP, complement control protein domain.

We performed database searches with the human gene sequences of CD248 and identified two orthologs in the zebrafish genome. Zebrafish *cd248a* and *cd248b* contained one single exon similar to that of other species. Zebrafish Cd248 shared similar protein domains as that of human and mouse. The deduced Cd248a had a single peptide, followed by seven conserved functional domains: one CLECT, one CCP domain, five EGF domains and a TM (transmembrane) domain. While the encoded Cd248b consisted a TM domain at the N-terminal, one CLECT domain, five EGF domains and a TM domain in the C-terminal (**Figure 1**). However, there was no CCP domain in Cd248b, which may be because of its loss during genome duplication. CLECT is C-type lectin domain or carbohydrate-recognition domain. The CLECT is a key structural motif responsible for assisting CD248 to recognize multiple ligands and functions to in defense against pathogens, cell trafficking, and immune regulation. EGF is Epidermal growth factor-like domain. EGF repeats mainly contribute to the interaction of EGF-containing protein with other proteins. CCP is complement control protein domain (also known as short consensus repeats SCRs or SUSHI repeats), CCP domain containing proteins are usually involved in complement pathway (7, 26).

**Figure 2:**
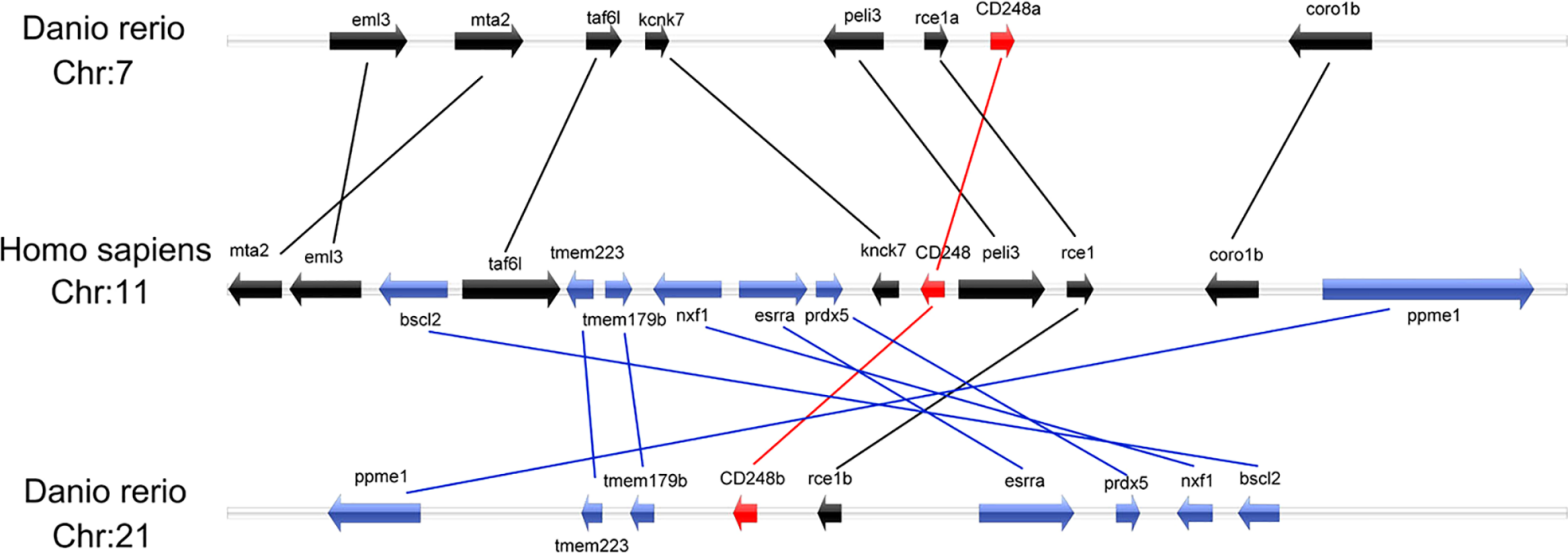
Colinearity analysis between zebrafish *cd248a*, *cd248b* and human *CD248*. All genes were indicated by arrows, and the arrow direction represented the gene direction. Silver strip, chromosome. Chr, chromosome.

To explore the possible synteny relationship among the zebrafish *cd248* and the human *CD248*, we analyzed genes surrounding the *cd248* loci in the zebrafish and human genomes. While *cd248a* was located on chromosome 7, *cd248b* was mapped to chromosome 21. The human *CD248* gene resided on chromosome 11. The analysis of the surrounding regions revealed that *cd248a* and 7 other zebrafish genes (*eml3, mta2, taf6l, kcnk7, peli3, rce1a and coro1b*) located on chromosome 7 had orthologs on human chromosome 11. Similarly, in the proximity of the *cd248b* locus, 7 zebrafish genes (*ppme1, tmem223, rce1b, esrra, prdx5, nxf1 and bscl2*) had their orthologs located near the human *CD248* locus on chromosome 11 (**Figure 2**). This conserved synteny strongly suggested that *cd248a* and *cd248b* were co-orthologs of the human *CD248* gene. *cd248a* and *cd248b* were originated from the presumed teleost-specific whole genome duplication event during evolution (27, 28).

### Phylogenetic analysis of cd248

**Figure 3:**
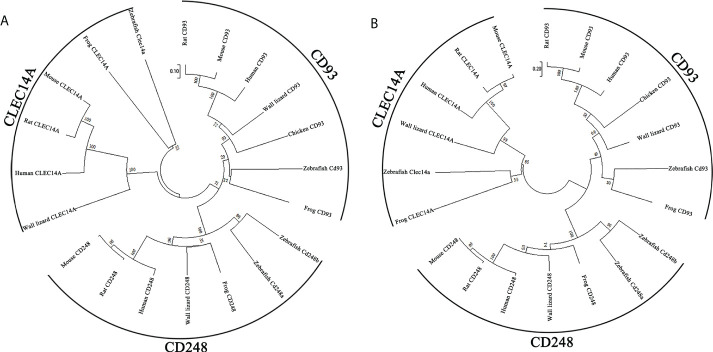
Phylogenetic trees of Cd248a and Cd248b. Phylogenetic trees constructed from the amino acid sequences of homologous genes of various species. CD248, CD93 and CLEC14A are members of the C-type lectin group 14 family. **(A)** neighbor-joining method; **(B)** maximum-likelihood method. The reliability of each node was estimated by bootstrapping with 1000 replications. The numbers shown at each node indicate the bootstrap values (%).

CD248 is also a member of C-type lectin domain group 14 family. To determine the relationship between zebrafish Cd248 and corresponding orthologues in different taxa, we conducted a phylogenetic analysis of the predicted protein sequences of C-type lectin domain group 14 family genes across 7 species (human, rat, mouse, frog, chicken, wall lizard and zebrafish) using the neighbor-joining method (**Figure 3A**) and maximum-likelihood method (**Figure 3B**). The phylogenetic trees showed that CD248, CD93 and CLEC14A orthologues among various species clustered together and divided into three distinct clades. As expected, zebrafish Cd248a and Cd248b formed a more closely branch, which further supported they were generated by teleost-specific genome duplication.

### Temporospatial expression of cd248

**Figure 4:**
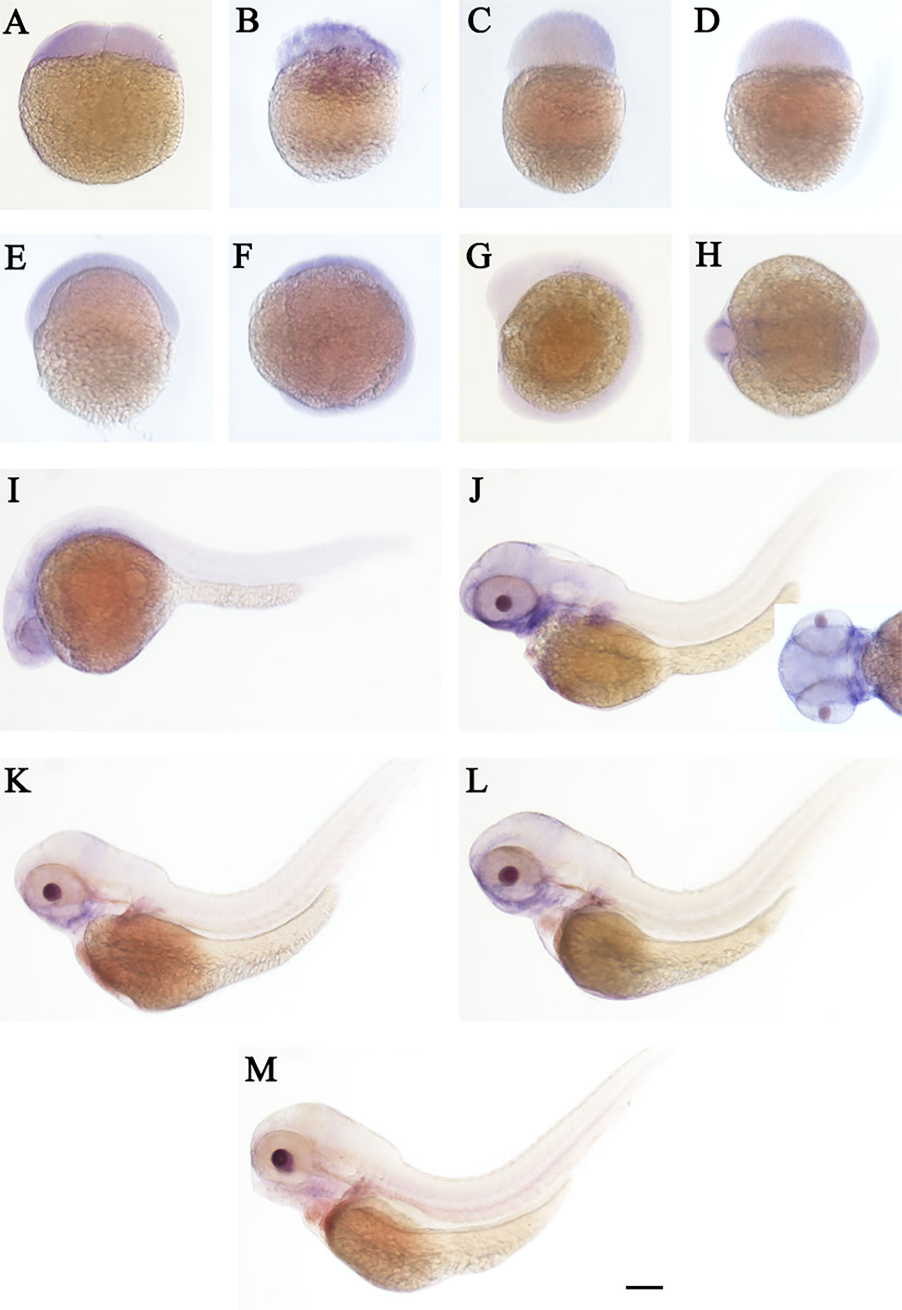
WISH of *cd248a* in zebrafish early embryos. Stages of embryonic development: **(A)** 2-cell; **(B)** 32-cell; **(C)** 512-cell; **(D)** sphere; **(E)** shield; **(F)** bud; **(G, H)** 10 somites; **(I)** 24 hpf; **(J)** 48 hpf; **(K)** 72hpf; **(L)** 96 hpf; **(M)** 120 hpf. hpf, hours post-fertilization. Scale bar, 100 μm.

**Figure 5:**
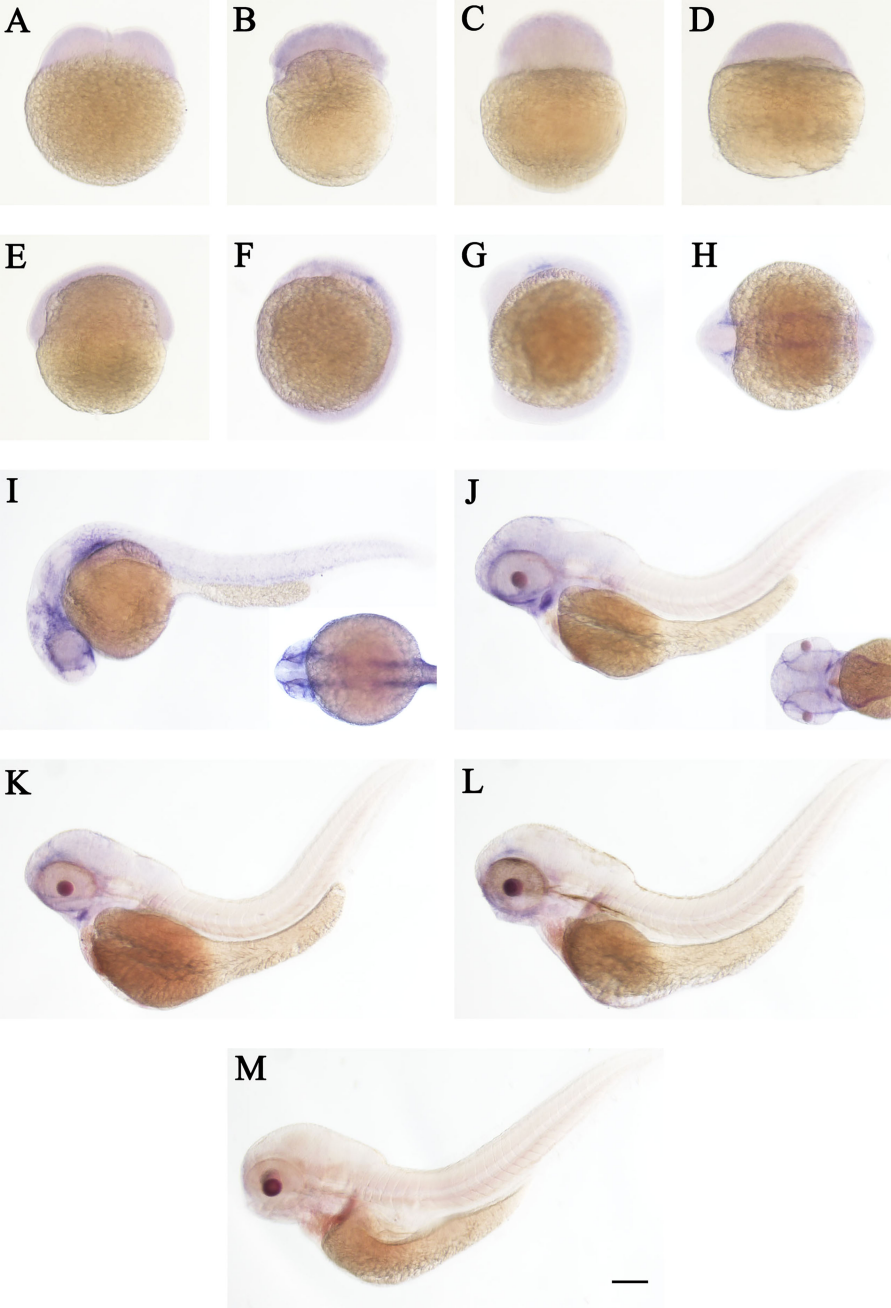
WISH of *cd248b* in zebrafish early embryos. Stages of embryonic development: **(A)** 2-cell; **(B)** 32-cell; **(C)** 512-cell; **(D)** sphere; **(E)** shield; **(F)** bud; **(G, H)** 10 somites; **(I)** 24 hpf; **(J)** 48 hpf; **(K)** 72hpf; **(L)** 96 hpf; **(M)** 120 hpf. hpf, hours post-fertilization. Scale bar, 100 μm.

The spatial and temporal expression patterns of *cd248a* and *cd248b* were determined by whole-mount *in situ* hybridization at early stages. *cd248a* and *cd248b* exhibited similar expression patterns. *cd248a* had weak maternal expression (**Figures 4A–F**). Zygotic transcript of *cd248a* were first seen in anterior somites and head mesenchyme at 14 hpf (**Figures 4G, H**); By 24 hpf, the expression was mainly detected in eyes and head mesenchyme (**Figure 4I**); From 48 to 120 hpf, *cd248*a showed a gradually declined expression pattern, the signal remained in the eyes, head mesenchyme, Additional expression was also observed in the branchial arches (**Figures 4J–M**). Weak ubiquitous maternal expression was also seen for *cd248b* (**Figures 5A–E**). The expression was observed in anterior somites and head mesenchyme at 10-14 hpf (**Figures 5F–H**), then shifted to eyes and head mesenchyme from 24 hpf on (**Figure 5I**); At 48hpf, the expression remained in eyes, head mesenchyme, and also expanded to branchial arches. At later stage, the expression signal gradually declined (**Figures 5J–M**).

### Expression responses of cd248 to LPS challenge

**Figure 6:**
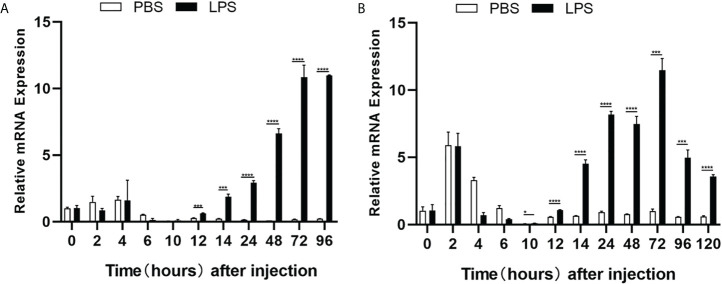
Quantitative analysis of zebrafish *cd248a* and *cd248b* genes in response to LPS/PBS treatment. **(A)** Quantitative analysis of zebrafish *cd248a* in infected embryos/larvae by microinjecting LPS at 1-cell stage; **(B)** Quantitative analysis of zebrafish *cd248b* in infected embryos/larvae by microinjecting LPS at 1-cell stage. PBS, Phosphate Buffered Saline; LPS, lipopolysaccharide. Data were shown as mean ± SD, *n* = 3. *P < 0.05; ***P < 0.001; ****P < 0.0001.

The expression profiles of zebrafish *cd248* in response to LPS challenge, which mimics infection of pathogenic bacteria, were examined. The dissociation curve of amplified products in all cases showed a single peak, indicating that the amplifications were specific (data not shown). Consistent with the expression pattern in untreated embryos, the mRNA expression level of *cd248a* in PBS injection group decreased gradually with development, excepted for a slightly higher expression level at 2-4 hours post-injection (hpi) (**Figure 6A**). By contrast, the mRNA expression of *cd248* were upregulated after LPS challenge. The mRNA expression level of *cd248a* increased soon after injection (2-4 hpi), and decreased at 4-10 hpi, then the expression level increased dramatically at 10-120 hpi (**Figure 6A**). The expression level of *cd248b* was highest in 2 hpi, decreased in 2-10 hpi, then remained low in 10-120 hpi (**Figure 6B**). Upon LPS challenge, the expression level of *cd248b* decreased in 2-10 hpi and then increased significantly in 10-120 hpi. Taken together, these data demonstrated that zebrafish early embryos/larvae were immunocompetent to LPS challenge *via* upregulating the expression of *cd248*.

### Cd248 localized on the cell membrane and cytoplasm

**Figure 7:**
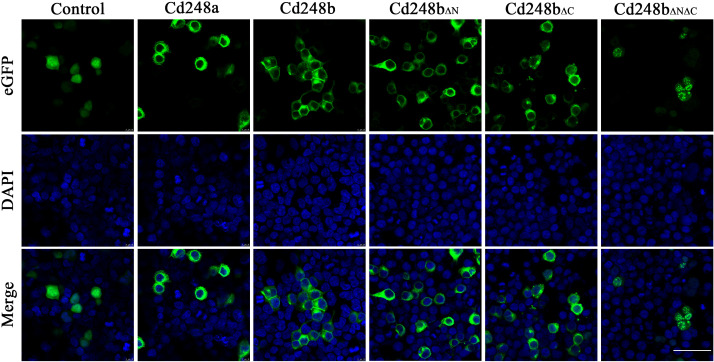
Subcellular localization of Cd248a, Cd248b and truncated Cd248b in HEK293T cells. The *pcDNA3.1/V5/Cd248a/eGFP, pcDNA3.1/V5/Cd248b/eGFP* and *pcDNA3.1/V5/eGFP* recombinant plasmids were transfected individually into HET293T cells. The nucleus was stained by DAPI. One representative image for each out of three independent experiments is shown. Scale bar, 50 μm.

To explore subcellular localization of Cd248a and Cd248b, the *pcDNA3.1/V5/cd248a/eGFP*, *pcDNA3.1/V5/cd248b/eGFP*, *pcDNA3.1/V5/cd248b_ΔN_/eGFP*, *pcDNA3.1/V5/cd248b_ΔC_/eGFP*, *pcDNA3.1/V5/cd248b_ΔNΔC_/eGFP* and *pcDNA3.1/V5/eGFP* (control) plasmids were transfected individually into HEK293T cells and stained DAPI at 24h post-transfected. As shown in **Figure 7**, eGFP was evenly distributed throughout the cell. In contrast, the green fluorescence in both Cd248a-eGFP and Cd248b-eGFP were observed in the periphery of nuclei, indicating they were localized on the cell membrane and cytoplasm. The subcellular localization of Cd248 was consistent with the predicted structures with a transmembrane region. It is striking to note that Cd248b lacking of N- or C-terminal transmembrane region still mainly localized on cell membrane and some in cytoplasm. By contrast, Cd248b lacking both transmembrane regions no longer localized on cell membrane, but in cytoplasm and nuclei as speckles.

### Effects of ectopic overexpression of Cd248 on production of pro-inflammatory cytokines in macrophages

**Figure 8:**
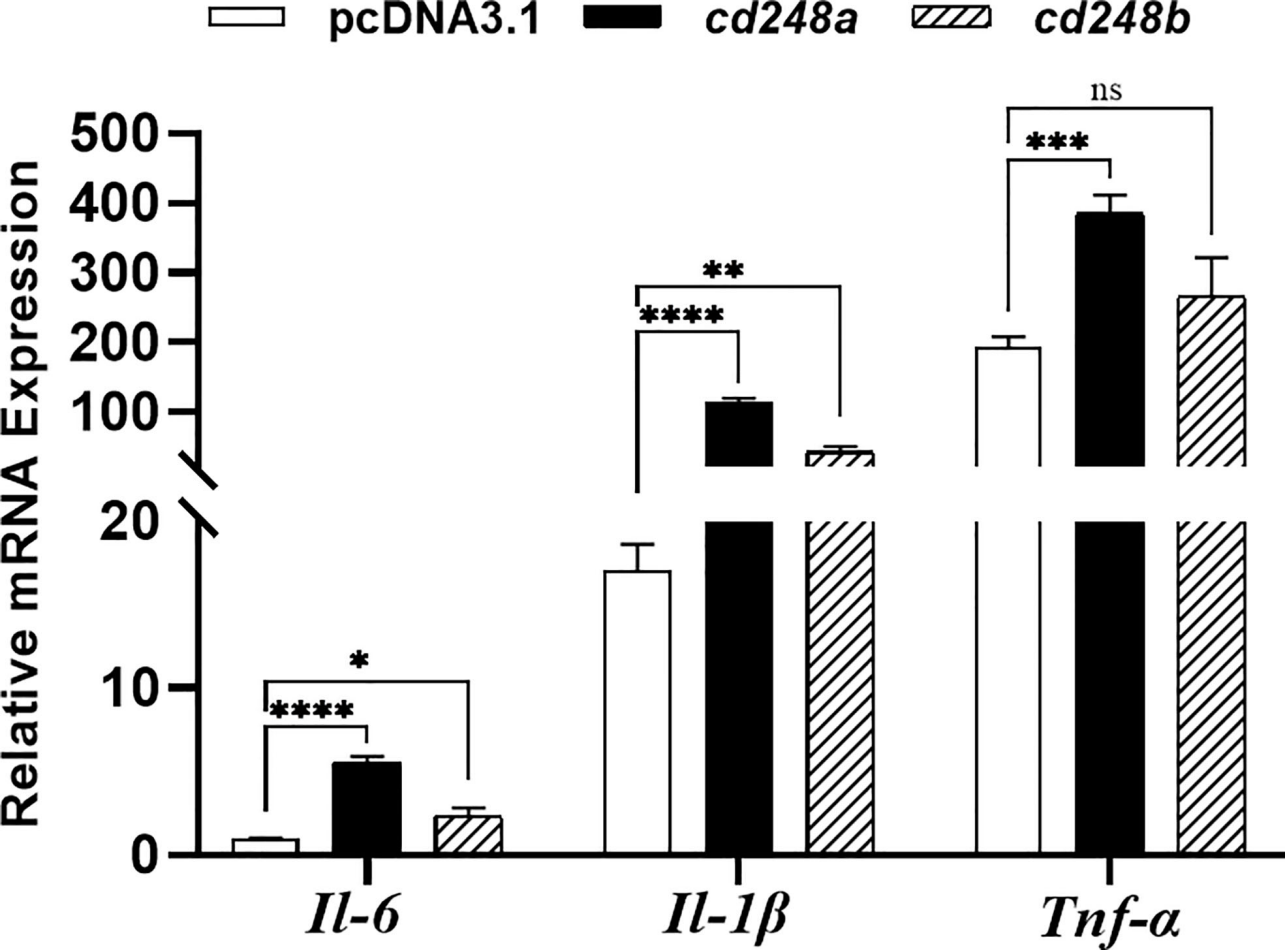
Effect of overexpression of zebrafish *cd248a* and *cd248b* on pro-inflammatory cytokines expression in mouse macrophages. The *pcDNA3.1/V5/Cd248a, pcDNA3.1/V5/Cd248b* and *pcDNA3.1/V5-His A* recombinant plasmids were transfected individually into RAW264.7 cells. At 24h after transfection, the total RNA of the cells was extracted and cDNA was synthesized. Quantitative analysis of the mRNA expression of mouse *Tnfα*, *Il1β* and *Il6*. Data were shown as mean ± SD. *P < 0.05; **P < 0.01; ***P < 0.001; ****P < 0.0001. ns, not significant.

Activated macrophages secrete various pro-inflammatory cytokines, such as *Il6*, *Il1β*, *Tnfα*, these cytokines cause a series of inflammatory reactions (29). Therefore, we sought to determine whether overexpression of *cd248a* and *cd248b* regulated the expression of pro-inflammatory cytokines in macrophages. To do this, *pcDNA3.1/V5/cd248a*, *pcDNA3.1/V5/cd248b* and *pcDNA3.1/V5-HisA* (control) plasmids were transfected individually into RAW 264.7 cells, and then the expression levels of *Il6*, *Il1β*, *Tnfα* were detected by qRT-PCR. As shown in **Figure 8**, the expression levels of *Il6*, *Il1β*, *Tnfα* were significantly upregulated by overexpression of *cd248a* and *cd248b*, suggesting that overexpression of *cd248a* and *cd248b* increased production of pro-inflammatory mediators in macrophages.

### Effects of ectopic overexpression of cd248 on production of pro-inflammatory cytokines *in vivo*

**Figure 9:**
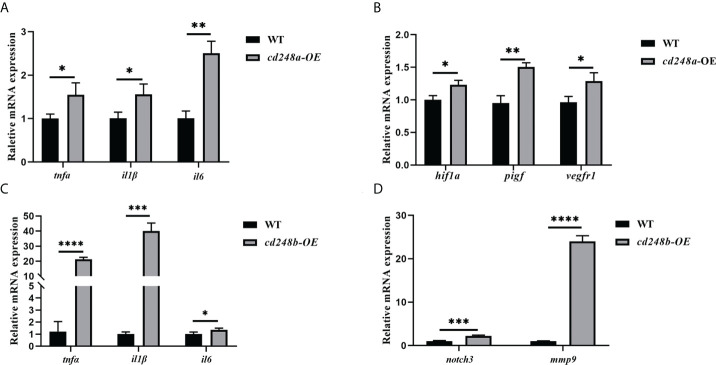
Effect of overexpression of zebrafish *cd248a* and *cd248b* on pro-inflammatory cytokines expression *in vivo*. Diluted mRNAs were injected into one-cell stage embryos of zebrafish. At 48h after injection, the total RNA of the embryos was extracted and cDNA was synthesized. **(A, C)** Quantitative analysis of the expression of *tnfα*, *il1β* and *il6* after *cd248a* and *cd248b* overexpression in embryos; **(B)** Quantitative analysis of the expression of *hif1a*, *pigf* and *vegfr1* after *cd248a* overexpression in embryos; **(D)** Quantitative analysis of the expression of *notch3* and *mmp9* after *cd248b* overexpression in embryos. *cd248a*-OE, *cd248a* overexpression; *cd248b*-OE, *cd248b* overexpression. Data were shown as mean ± SD. *P < 0.05; **P < 0.01; ***P < 0.001; ****P < 0.0001.

Overexpression of *cd248a* and *cd248b* could significantly increase the expression of pro-inflammatory cytokines *in vitro*. To investigate whether overexpression of *cd248* has the same effects *in vivo*, we overexpressed *cd248a* and *cd248b* mRNA in zebrafish embryos and verified that they can significantly increase the expression of pro-inflammatory cytokines *in vivo* (**Figures 9A, C**). It has been well established that the activation of Notch signal could increase the expression of MMP9 and further increase the expression of inflammatory cytokines (30–33). Our results showed that overexpression of *cd248b* increased the expression of *notch3* and *mmp9* (**Figure 9D**), but that was not the case for *cd248a* overexpression, which suggested the upregulation of expression pro-inflammatory cytokines upon *cd248a* overexpression was not regulated by Notch signaling pathway (**Figure S1A**). We noted VEGF (Vascular endothelial growth factor) signaling pathway is a major regulator of vascular development and lymphatic function, which also plays an important role in tumor growth, retinopathy, tissue inflammation and immunity (34, 35). In this pathway, HIF1A acts as a central link and transmits signals to the downstream PIGF (36–39). PIGF (placental growth factor), is a member of the VEGF family, which can bind VEGFR1 with high affinity, plays a key role in pathological angiogenesis, especially in cancer, cardiovascular, autoimmune and inflammatory diseases (40, 41). Thus, we examined the expression of VEGF signals upon *cd248a* overexpression, as expected, overexpression of *cd248a* increased the expression of *hif1a* and also upregulated the expression of *pigf* and *vegfr1*(**Figure 9B**). However, *cd248b* overexpression decreased the level of *hif1a* (**Figure S1B**). Taken together, the overexpression of *cd248a* and *cd248b* upregulated the expression of pro-inflammatory cytokines *in vivo* by different mechanisms, possibly *via* VEGF pathway and Notch pathway respectively. These findings suggested that zebrafish *cd248a* and *cd248b* participated in the immune response.

### Deficiency of cd248 downregulated the expression of pro-inflammatory cytokines and upregulated anti-inflammatory cytokines

**Figure 10:**
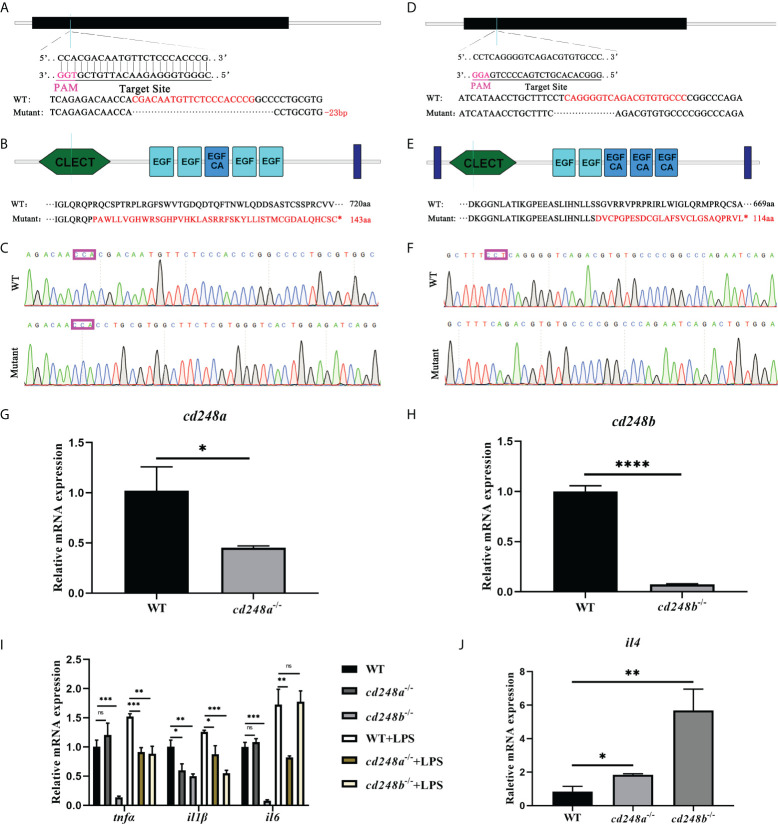
Effect of *cd248* deletion on inflammatory cytokines. The mutants of *cd248a* and *cd248b* were carried out by CRISPR/Cas9 system. **(A–C)** CRISPR/Cas9 gRNA design and the genotyping result of the *cd248a*-mutant allele; **(D–F)** CRISPR/Cas9 gRNA design and the genotyping result of the *cd248b*-mutant allele; **(G, H)** Quantitative analysis of the expression of *cd248a or cd248b* after *cd248a*-deletion or *cd248b*-deletion compared with WT respectively; **(I)** Quantitative analysis of the expression of zebrafish *tnfα*, *il1β* and *il6* in the *cd248*-mutants and LPS treatment. **(J)** Quantitative analysis of the expression of *il4* in the *cd248*-mutants compared with WT. Data were shown as mean ± SD. *P < 0.05; **P < 0.01; ***P < 0.001; ****P < 0.0001. ns, not significant.

To better investigate the role of *cd248*, we generated *cd248a*-null mutant and *cd248b*-null mutant with 23 bp and 10 bp deletions by using the CRISPR/Cas9 system respectively (**Figures 10A, B, D, E**). The two mutations both caused frameshift in the protein-coding region leading to early termination of translation (**Figures 10C, F**). Both mRNA level downregulated dramatically in *cd248* mutants compared with WT (**Figures 10G, H**). As described above, overexpression of *cd248* could upregulate the expression of pro-inflammatory cytokines, we wondered the effects of impaired *cd248* function on the expression of pro-inflammatory cytokines. As expected, both *cd248* deletions reduced the expression of pro-inflammatory cytokines (**Figure 10I**), although no significant difference of the level of *tnfα* and *il6* observed between *cd248a* mutant and WT (**Figure 10I**).

Even after LPS treatment, the expression of pro-inflammatory cytokines in the mutant were lower than that in the WT (**Figure 10I**). In addition, deficiency of *cd248a* and *cd248b* both upregulated the expression of anti-inflammatory factor *il4* compared with WT (**Figure 10J**). Furthermore, the level of VEGF signals in *cd248a* mutant was comparable with WT (**Figure S2A**), whereas the expression of Notch signals was reduced in *cd248b* mutant than WT (**Figure S2B**). Collectively, these results further confirmed that Cd248 proteins were involved in the innate immune process.

## Discussion

CD248 has gained wide attention due to the relevance in human health and disease, particularly in the fields of innate immunity and inflammation (26). However, no definite *cd248* orthologs in fish have been documented so far. In this study, we reported the identification of *cd248* in zebrafish *D. rerio*, the first *cd248* orthologs in fish.

Zebrafish *cd248a* and *cd248b* are orthologs of human *CD248* supported by several lines of evidences. Both *cd248a* and *cd248b* in zebrafish are intronless, like CD248 homologs in human and mice (15). Zebrafish Cd248 proteins shared similar protein domains as that of other species. No CCP domain was found in human CD248 by SMART domain prediction online tools, although it is reported that a CCP domain is presented in human CD248. The discrepancy may be caused by different tools and different algorithms. In addition, the CCP domain also exists in CD248 of rat, wall lizard, and clawed frog by SMART domain prediction online tools. However, there was no CCP domain in Cd248b, which may be because of its loss during genome duplication. Moreover, there was no CCP domain in Cd248 of medaka, but CCP domain exists in Cd248-like of medaka. This conclusion further confirmed that one of Cd248 lost the CCP domain in the process of fish genome duplication. In humans, the *CD248* is located on chromosome 11, while zebrafish *cd248a* and *cd248b* are located on chromosome 7 and 21, respectively. Despite the order and direction are different, the genes upstream and downstream of *CD248* displayed fine synteny in zebrafish and human. This conserved synteny strongly suggested that *cd248a* and *cd248b* are co-orthologs of the human *CD248* gene. Alignment with those of other group XIV CTLD-containing proteins revealed that Cd248 shared the highest homology to CD248 (Data not shown). The two independent phylogenetic trees have firmly clustered the zebrafish Cd248a and Cd248b with other vertebrate CD248 homologs. In addition, zebrafish Cd248a and Cd248b shared highly homology, and formed a more closely branch in the phylogenetic tree, which further supported they were generated by teleost-specific genome duplication. We failed to identify authentic CD248 orthologs in invertebrates (such as *Amphioxus*, *Drosophila*, *Nematodes*), CD248 is a member of the group XIV of C-type lectin domain-containing proteins (CTLDcps). CTLDcps appeared with the advent of early vertebrates after a whole genome duplication followed by a sporadic tandem duplication (42). Therefore, we speculated CD248 were arisen only in the vertebrate lineage. It is interesting to note that chicken CD248 (according to NCBI database) clustered together with other species CD93, thus we determined chicken CD248 is actually CD93, not a CD248 ortholog, this is consistent with reported that the losing CD248 in birds was due to chromosomal breakage (42).

Human CD248 is known to be a single transmembrane cell surface glycoprotein (4). The Domain prediction has shown Cd248a possesses a single transmembrane domain while Cd248b contains two transmembrane domains located near the N- and C-termini. The subcellular localization experiment has confirmed both Cd248a and Cd248b expressed on the cell membrane and cytoplasm. Dfi1 protein also contains two transmembrane domains. Similarly, Dfi1 lacking the N-terminal transmembrane region was observed at the cell periphery where full-length Dfi1 normally localizes, whereas the double-truncation mutant was diffusely intracellular (43). Further data suggested the two transmembrane domains contributed to the biogenesis of Dfi1 (43). Cd248b lacking either of transmembrane region still mainly expressed in the cell membrane and cytoplasm, whereas double transmembrane domains deletion mutant was localized in the cytoplasm and nuclei as speckles. These data suggested the two transmembrane domains possibly contributed to the biogenesis of Cd248b and the function of the two transmembrane domains is worthy of further exploration.

CD248 is predominantly expressed on stromal fibroblasts throughout the mesenchyme and on the developing vasculature in mice embryos. The expression of endosialin gradually downregulated during development resulting in great loss of expression in adult tissues (3). In this study, we confirmed that *cd248a* and *cd248b* exhibited similar expression patterns in zebrafish. Both genes had weak ubiquitous expression at early stage, the zygotic transcripts were first detected in the head mesenchyme and anterior somite, then mainly in the head mesenchyme, eyes and branchial arches, and gradually declined with development, implying that these two genes may be involved in the formation these tissues.

CD248 is a member of C-type lectin domain group 14 family, which also contains TM (thrombomodulin), CD93 and CLEC14A (6). To date, there are many members of C-type lectin family, served as pattern recognition receptors, play important roles in immune responses (24, 44–52). Loss-of-function studies in mice implied CD248 promoted tumor growth and inflammation (26). Zebrafish Cd248a and Cd248b, members of C-type lectin, were expressed on the cell membrane and cytoplasm, we wondered whether zebrafish Cd248 participated in the immune response. The mRNA expression of *cd248a* and *cd248b* were upregulated upon LPS treatment, suggesting *cd248a* and *cd248b* were involved in innate immunity of zebrafish. We found that overexpression of *cd248a* and *cd248b* both upregulated the expression of pro-inflammatory cytokines *in vitro and in vivo*. These results suggested that *cd248a* and *cd248b* were involved in innate immune responses. It is worth noting that *cd248a* upregulated the expression of pro-inflammatory cytokines possibly *via* the VEGF signaling pathway. But *cd248b* increased the expression of pro-inflammatory cytokines possibly *via* Notch signaling pathway. Although our study was conducted in zebrafish embryos, and we confirmed overexpression and knockout of *cd248a* or *cd248b* influenced on the expression of pro-inflammatory cytokines. We found the link of pro-inflammatory cytokines and VEGF signal or Notch signal in the references, although the data were derived from disease and immunity. Interestingly, in the transcriptomic data of RNA-seq, we noted VEGF signal in *cd248a* mutant, Notch signal in *cd248b* mutants changes compared to that in Wild type (Data not shown). Hence, we examined VEGF signal or Notch signal and found that overexpression and knockout of *cd248a* changed VEGF signal but not Notch signal, overexpression and knockout of *cd248b* changed Notch signal but not VEGF signal. Therefore, we speculated that *cd248a* and *cd248b* promoted pro-inflammatory cytokines possibly *via* these two signaling pathways respectively. It was uncertain whether *cd248a* or *cd248b* directly acts through VEGF signaling pathway or Notch signaling pathway on pro-inflammatory cytokines in zebrafish. It will be interesting to elucidate whether there were other molecules involved and whether other signaling pathways were participated. It was previously reported that human CD248 inhibits the Notch signaling pathway and then activates the expression of pro-inflammatory cytokines (53), which was inconsistent with our conclusion that *cd248b* activated the Notch signaling pathway and then activated the expression of pro-inflammatory cytokines. In fact, it has been reported that inhibiting Notch signaling pathway promotes the expression of pro-inflammatory cytokines (54), and others reported that activating Notch signaling pathway promotes the expression of pro-inflammatory cytokines (55–60). Different conclusions may be due to different functions of CD248 in different species. In mice, fibronectin and type I and V collagen have been identified as specific ligands of CD248 (1). CD248 promotes inflammation possibly *via* intracellular signaling *via* its cytoplasmic tail and/or through extracellular matrix ligands that interact with the ectodomain (15). In zebrafish, to identify the specific ligands of Cd248 protein may help to elucidate how they participated in innate immune responses.

The zebrafish *cd248* mutants had no visible phenotype, and we did not observe any cartilage defects by Alcian Blue staining compared with WT (data not shown). Regrettably, we had no specific antibodies against Cd248a protein or Cd248b protein to verify the experimental results at the protein level in the mutants. We only examined the mRNA expression level of the mutants, and the results showed that mRNA levels of *cd248a* and *cd248b* were decreased. The absence of *cd248a* and *cd248b* both downregulated expression of pro-inflammatory cytokines, but the regulatory level of *cd248a* was weaker than that of *cd248b*, which is in part because the more decreased of mRNA level of *cd248b* in the mutant. Additionally, we will try to obtain the double-mutant of zebrafish *cd248* in order to better elucidate the role of Cd248 protein in immune system.

In summary, we identified two orthologs of CD248, *cd248a* and *cd248b*, in zebrafish. The duplicated *cd248* resemble the mammalian counterpart and exhibited similar expression pattern. Both *cd248a* and *cd248b* could be upregulated by LPS challenge. Moreover, overexpression of *cd248a* or *cd248b* promoted the production of pro-inflammatory cytokines. Loss-of-function suggested that zebrafish *cd248a and cd248b were* involved in innate immune process. However, the possible ligands of Cd248 haven’t been elucidated. Additionally, whether the duplicated Cd248 were redundantly involved in early development remains to be further investigated.

## Data availability statement

The original contributions presented in the study are included in the article/**Supplementary Material**. Further inquiries can be directed to the corresponding authors.

## Ethics statement

This study was reviewed and approved by Animal Committee of Ocean University of China.

## Author contributions

All authors have read and approved the manuscript. XL and RG performed the experiments, wrote and revised the manuscript. SY, XZ, XY, LT, SZ analyzed the data. GJ revised the manuscript. HL designed the experiments and revised the manuscript. All authors contributed to the article and approved the submitted version.

## Funding

This work was supported by the grants (31872187, 32171139) of Natural Science Foundation of China (NSFC) and grant (2018YFD0900502) of the Ministry of Science and Technology (MOST) of China.

## Conflict of interest

The authors declare that the research was conducted in the absence of any commercial or financial relationships that could be construed as a potential conflict of interest.

## Publisher’s note

All claims expressed in this article are solely those of the authors and do not necessarily represent those of their affiliated organizations, or those of the publisher, the editors and the reviewers. Any product that may be evaluated in this article, or claim that may be made by its manufacturer, is not guaranteed or endorsed by the publisher.
