# Supplementary material for: Cd248a and Cd248b in zebrafish participate in innate immune responses
Source: Front Immunol. 2022 Aug 31;13:970626. doi: 10.3389/fimmu.2022.970626 (PMC9471012; doi:10.3389/fimmu.2022.970626)
Supplement: Supplementary file 1 [file DataSheet_1.docx]

**
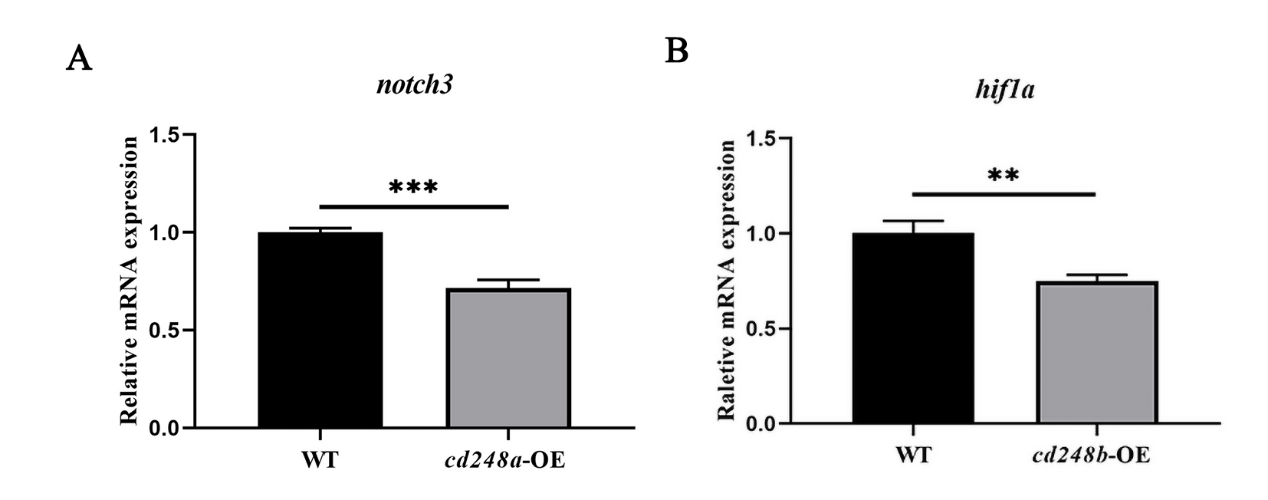
**

**Fig. S1. The effect of overexpression of *cd248a* or *cd248b* on *notch3* or *hif1a*.**

A, Quantitative analysis of the expression of *notch3* in the *cd248a* overexpression compared with WT; B, Quantitative analysis of the expression of *hif1a* in the *cd248b* overexpression compared with WT. Data were shown as mean ± SD. ***P* < 0.01; ****P* < 0.001.


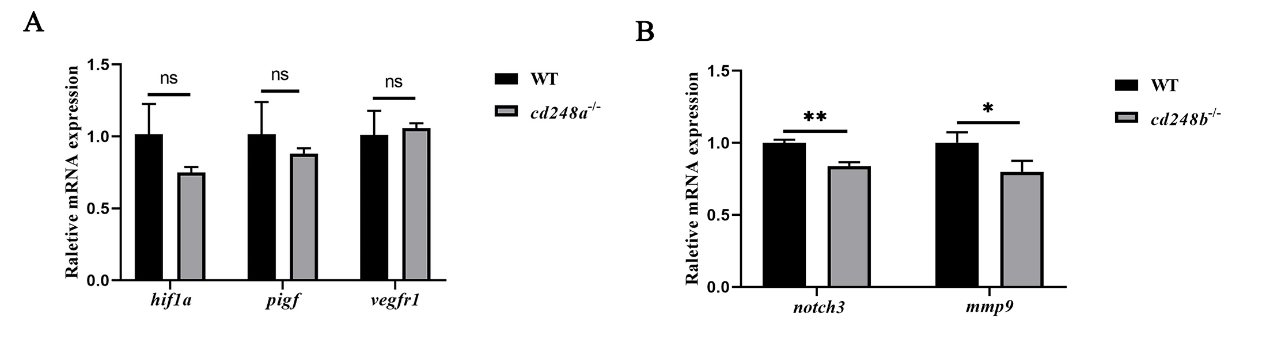


**Fig. S2. Loss of *cd248a* or *cd248b* affect the expression of VEGF signals or Notch signals.**

A, Quantitative analysis of the expression of *hif1a* in the *cd248a*-mutant compared with WT; B, Quantitative analysis of the expression of *notch3* and *mmp9* in the *cd248b*-mutant compared with WT. Data were shown as mean ± SD. **P* < 0.05; ***P* < 0.01; ns, not significant.

**Supplemental Table 1: Primers used in this study.**

| Primers | Sequences (5’-3’) | | | Sequence information |
| --- | --- | --- | --- | --- |
| P1 (F)  P2 (R)  P3 (F)  P4 (R)  P5 (F)  P6 (R)  P7 (F)  P8 (R)  P9 (F)  P10 (R)  P11 (F)  P12 (R)  P13 (F)  P14(R)  P15 (F)  P16(R)  P17 (F)  P18(R)  P19 (F)  P20(R)  P21 (F)  P22(R)  P23(F)  P24(R)  P25(F)  P26(R)  P27(F)  P28(R)  P29(F)  P30(R)  P31(F)  P32(R)  P33(F)  P34(R)  P35(F)  P36(R)  P37(R)  P38(F)  P39(F)  P40(R)  P41(F)  P42(R)  P43(F)  P44(R) | | CCGGAATTCATGGGGTGTCCAGTTCTGTG CCGCTCGAGAACGTGGGACTTAGTTCCGC CCGGAATTCATGGGGAAAAGGGCTGATA  CCGCTCGAGAGCCTGAGATTTTGCTGTGCT TTTCCCACGGACCTGATT  GTGGGACTTAGTTCCGCTC ACATCGCTGCAAGTGCC  TTTTGTTTCCTGAAGCCCTA ATGAGGGTTTTCTCCTGGCTG  TCCAGGTGAATTTACACAGATATGC CACCAAGCATAGAGTGGCATC  CTTTTGTTTCCTGAAGCCCTA GGTATTGTGATGGACTCTGGTGAT  TCGGCTGTGGTGGTGAAG  CCGGAATTCATGGGGTGTCCAGTTCTGTG CCGCTCGAGTTAAACGTGGGACTTAGTTCCGC  CCGGAATTCATGGGGAAAAGGGCTGATA  CCGCTCGAGTCAAGCCTGAGATTTTGCTGTGCT  CAGGGGCCACCACGCTCTTC  CTTGGGGCAGGGGCTCTTGAC  GCCCATCCTCTGTGACTCAT  AGGCCACAGGTATTTTGTCG  TGCCTTCTTGGGACTGAT  CTGGCTTTGTCTTTCTTGTT  CTTGGGGCAGGGGCTCTTGAC  GCTGTGGTGGTGAAGCTGTA  GCTGGTGATGGTGTCTAGGAGGAA  CCCTGGGTCTTATGGAGCGTGAA  GTCTGCTACACTGGCTACACTCTTC  CGTCCACATCCTGAACTTCGTCTC  GTCTGCTACACTGGCTACACTCTTC  CGTCCACATCCTGAACTTCGTCTC  CCCACAGCGTCACCTCTAACCT  GCAGTCTCCTTCTCCAGCACATC  GCTCATGCCATTATCGCCTCCTC  CCAGCCACGCAATACAGCCAAA  GTCGGAGATCCCAGATGTGGTGTA  TGCCTGCTGTTCCAGATGATATTCC  GAAGGATTGCTCTGCGTGGATAC  CATTTAGACAAGGCGAACGGTG  TCGTTGAGAGCCTGGTGTTTGC  TGCCGCTGGAGCTTCTTCAGA  TGCCAAGCAGGAATGGCTTTGAA  TGCAGTTTCCAGTCCCGGTATATG | For recombinant expression  For recombinant expression  For recombinant expression  For recombinant expression  For WISH  For WISH  For WISH  For WISH  For qRT-PCR  For qRT-PCR  For qRT-PCR  For qRT-PCR  For qRT-PCR  For qRT-PCR  For recombinant expression  For recombinant expression  For recombinant expression  For recombinant expression  For qRT-PCR  For qRT-PCR  For qRT-PCR  For qRT-PCR  For qRT-PCR  For qRT-PCR  For qRT-PCR  For qRT-PCR  For qRT-PCR  For qRT-PCR  For qRT-PCR  For qRT-PCR  For qRT-PCR  For qRT-PCR  For qRT-PCR  For qRT-PCR  For qRT-PCR  For qRT-PCR  For qRT-PCR  For qRT-PCR  For qRT-PCR  For qRT-PCR  For qRT-PCR  For qRT-PCR  For qRT-PCR  For qRT-PCR | |

**Supplemental Table 2:** **NCBI Reference Sequences used in phylogenetic analyses.**

| Species | Proteins | NCBI Reference Sequences |  |
| --- | --- | --- | --- |
| Zebrafish | Cd248a | NP_001092698.3 |  |
|  | Cd248b | XP_021324763.1 |  |
|  | Cd93 | XP_005159016.1 |  |
|  | Clec14a | NP_956080.1 |  |
| Human | CD248 | NP_065137.1 |  |
|  | CD93 | NP_036204.2 |  |
|  | CLEC14A | NP_778230.1 |  |
| House mouse | CD248 | NP_473383.1 |  |
|  | CD93 | NP_034870.1 |  |
|  | CLEC14A | NP_080085.3 |  |
| Norway rat | CD248 | NP_001099795.1 |  |
|  | CD93 | NP_445835.1 |  |
|  | CLEC14A | NP_001014099.1 |  |
| Tropical clawed frog | CD248 | XP_004913746.1 |  |
|  | CD93 | XP_002936962.2 |  |
|  | CLEC14A | XP_002935391.1 |  |
| Common wall lizard | CD248 | XP_028566091.1 |  |
|  | CD93 | XP_028594219.1 |  |
|  | CLEC14A | XP_028576116.1 |  |
| Chicken | CD93 | NP_001186380.1 |  |
